# Supraglottic Foreign Body Missed for One Month in a Child

**DOI:** 10.7759/cureus.33870

**Published:** 2023-01-17

**Authors:** Mohamed Iliyas Sultan Abdul Kader, Nadia Syafeera, Khadijah Md Nor, Saraiza Abu bakar, Marina Mat Baki

**Affiliations:** 1 Department of Otorhinolaryngology-Head and Neck Surgery, Hospital Melaka, Melaka, MYS; 2 Department of Otorhinolaryngology-Head and Neck Surgery, Faculty of Medicine, Universiti Kebangsaan Malaysia Medical Centre, Kuala Lumpur, MYS; 3 Otolaryngology-Head and Neck Surgery, Universiti Putra Malaysia, Selangor‎, MYS; 4 Department of Otorhinolaryngology, Hospital Serdang, Serdang, MYS

**Keywords:** pediatric laryngology, delayed diagnosis., hoarseness, aspiration, foreign body

## Abstract

Foreign body (FB) aspiration in children can result in serious complications that can lead to even death. We present a case of a one-year-old girl child with a history of choking one month prior while she was feeding. A bolus of rice was removed at a local clinic. Consequently, within 24 hours, she developed hoarseness and noisy breathing which was treated as an upper respiratory tract infection at two different clinics. This case report aims to highlight the need for otolaryngology consultation in a child with non-resolving respiratory symptoms following episodes of choking. This will prompt an immediate surgical intervention that could prevent potential morbidity and mortality as a result of a compromised airway.

## Introduction

Foreign body (FB) aspiration results in mechanical suffocation and remains one of the leading causes of preventable deaths in infants. In the United States alone, children who are 5 years of age and younger account for close to seventy-thousand FB ingestions annually [1]. Malaysian children observed a peak incidence of FB in the age groups between 12 and 24 months. Children in this age group are more aware of their surroundings and the child’s ability to move around increases [2].

Chen et al. retrospectively analysed 220 children with FB and found about 54% of them had delayed diagnosis [3]. The vast majority of FB aspiration events remain nonfatal. However, airway FB can be life-threatening; the rate of death or anoxic brain injury associated with paediatric FB is approximately 4% [4]. Morbidity and financial costs of FB aspiration are still a problem in both developed and underdeveloped countries [4].

## Case presentation

A one-year-old girl had a choking episode one month prior to the presentation, while she was being fed rice by her mother. She was immediately brought to a local clinic. A rapid and thorough assessment was done, followed by the successful manual removal of a bolus of rice. She was clinically well post-procedure and was discharged. She developed hoarseness the following day and concomitant progressive stridor. Subsequently, two visits were made to different clinics where she was treated for coryza and viral-induced wheezing; she was prescribed bronchodilators and antihistamines. Despite having stridor, she remained afebrile and was comfortable in room air. However, the hoarseness and stridor worsened over one month, leading to a referral to an otorhinolaryngology (ORL) clinic.

Clinically, there was an inspiratory stridor and dysphonia at rest with a marked component of roughness. She was able to tolerate bottle feeding with no aspiration. She was afebrile and not in respiratory distress. There was no neck swelling or limited neck movements, there were no intercostal and subcostal recessions; lungs examination revealed no abnormality. Plain chest and lateral neck radiographs were normal. In-office flexible nasopharyngolaryngoscopy revealed a FB with granulation tissue at the level of the supraglottis.

**Figure 1:**
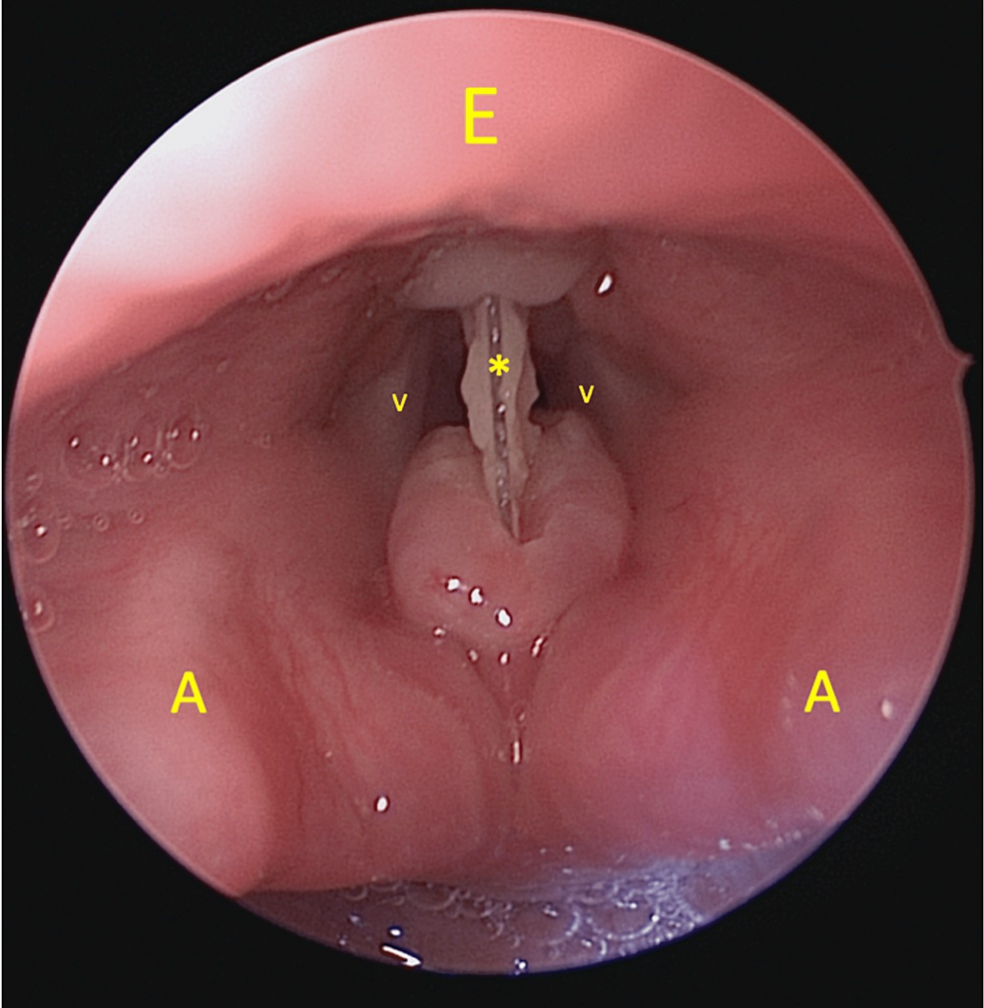
Direct laryngoscopy shows a foreign body stuck at the level of false fold, traversing from its anterior to posterior walls. A: Arytenoids, E: Epiglottis, V: Vocal folds, *:Foreign body.

**Figure 2:**
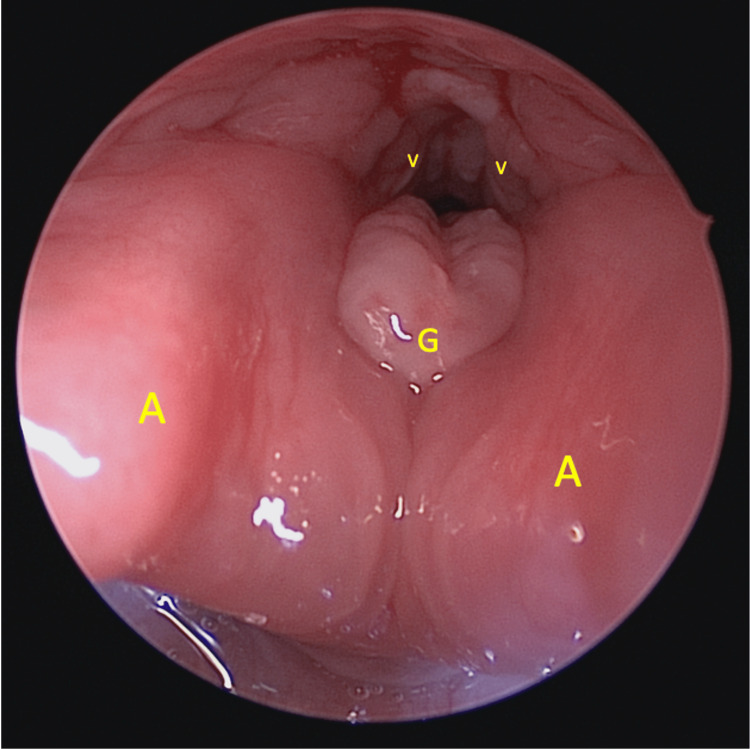
Granulation tissue at the anterior surface of the arytenoid not extending to the vocal folds. A: Arytenoids, V: Vocal folds, G: Granulation tissue.

**Figure 3:**
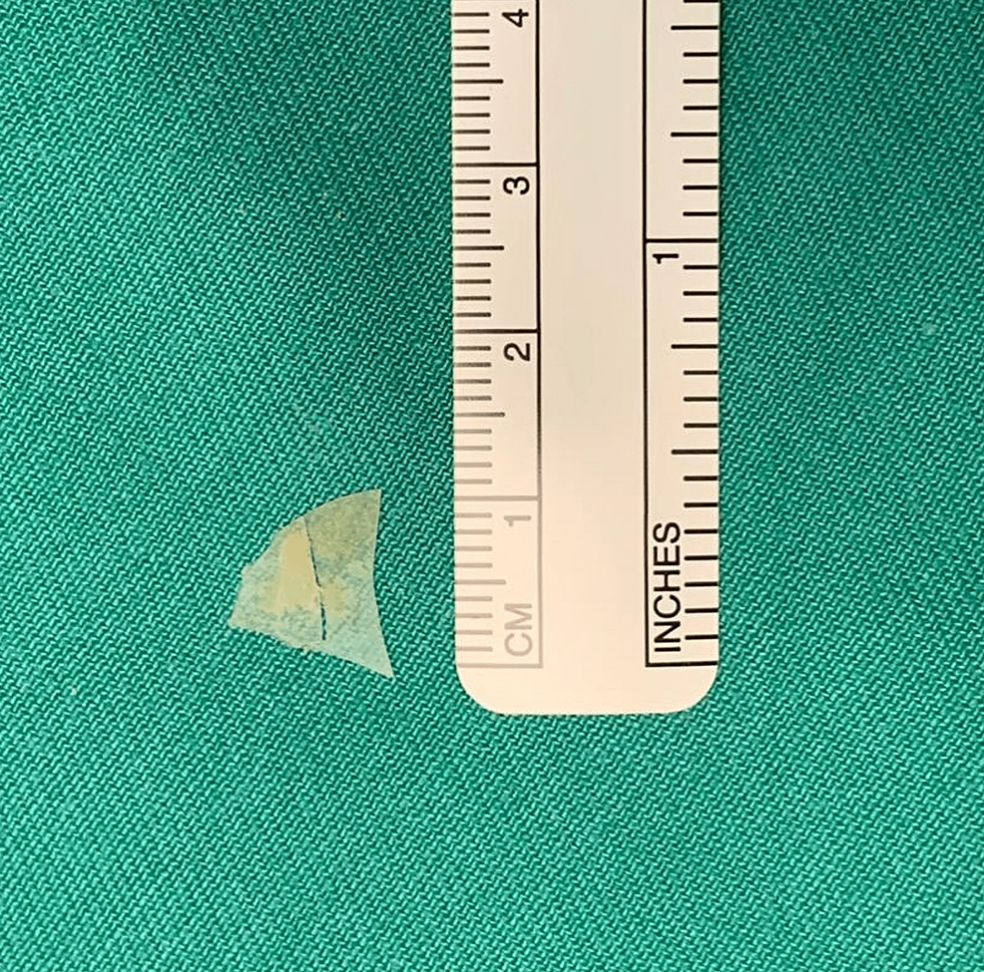
A single piece of triangular plastic material removed measuring 1 cm x 1 cm.

She underwent an emergency examination under general anaesthesia through spontaneous ventilation via inhalational induction. The FB was seen to be embedded at the level of the false fold, traversing from its anterior to posterior walls (Figure 1). There was also granulation tissue at the anterior surface of the arytenoid of the posterior commissure and at the laryngeal surface of epiglottis around the petiole area, due to mucosal reaction towards the FB (Figure 2). There was no extension of granulation tissue to the true vocal folds. One piece of hard plastic material measuring 1 x 1 cm was removed (Figure 3).

The second inspection post removal of FB using a flexible bronchoscope did not reveal any other FB in the distal airway. Intravenous dexamethasone (0.1 mg/kg) was given to reduce the risk of potential airway oedema. Anaesthesia was subsequently reversed, and the child was kept in the paediatric high-dependency unit for observation followed by observation in the normal ward prior to discharge. She remained well and was eventually discharged.

## Discussion

Infants and toddlers tend to put everything in their mouths. Their neural coordination of swallowing and breathing is still developing. In addition, the majority of the children are often playing, running, or laughing while eating, and this explains the higher prevalence of inhaled or aspiration of FB [2]. FB aspiration can greatly masquerade as other respiratory tract diseases due to their non-specific symptoms. This can pose substantial challenges in diagnosis even among experts, causing further intervention delays. A meta-analysis showed that the diagnosis of inhaled FB was delayed by more than 24 hours in almost 40% of the cases [5].

In our case, the laryngeal function was compromised for a month due to the remaining FB being missed during the initial diagnosis. Three clinical phases were identified in FB aspiration. First phase shows choking, gagging, and bouts of coughing occurring at the time of ingestion. These can cause sudden death due to asphyxia, but the majority of the time, these symptoms resolve as reflexes slow down thus leading to the second phase or asymptomatic phase. In this phase, FB lodges in the airway and causes ambiguous respiratory symptoms which can last for a long term, Third phase or complication phase is when FB causes obstruction, erosion or infection which can then results in drug-resistant pneumonia, atelectasis, bronchiectasis, pneumothorax, mediastinum abscess, perforation or erosion leading to bronchoesophageal fistula [6,7].

During the first visit, the local clinic managed to remove a rice bolus. Post removal, the patient was asymptomatic and he was discharged. However, the child developed respiratory symptoms and later sought treatment at two different clinics. Respiratory symptoms that develop in a child with a previous history of aspiration despite removal should raise the suspicion of residual or second FB in the airway, hence urgent referral to ORL should be made. Chest and lateral neck radiographs offered little diagnostic value in this case, probably due to the radiopaque nature of the ingested FB. Our case highlights that a FB left in the airway can remain clinically silent or have symptoms that may mimic other airway diseases. Not all FB aspiration will show marked symptoms of respiratory distress as the FB might not obscure the whole airway.

FB in the lower airway can increase the risk of recurrent pneumonia and often presents late, leading to a delayed diagnosis [8]. We believe that all cases of respiratory symptoms, following a history of choking after FB removal, should be referred to the ORL centre for a prompt airway assessment. This is important as residual FB can result in preventable life-threatening complications.

## Conclusions

History of a choking episode followed by non-resolving respiratory symptoms requires urgent referral to the ORL centre for further assessment. An emergency diagnostic laryngoscopy with bronchoscopy is pertinent to prevent catastrophic complications. Plain radiographs of the chest and neck do not rule out inhaled FB.
